# Platform-agnostic electrochemical sensing app and companion potentiostat

**DOI:** 10.1039/d2an01350a

**Published:** 2023-08-01

**Authors:** Vijayalaxmi Manoharan, Rui Rodrigues, Sara Sadati, Marcus J. Swann, Neville Freeman, Bowen Du, Ender Yildirim, Ugur Tamer, Theodoros N. Arvanitis, Dmitry Isakov, Ali Asadipour, Jérôme Charmet

**Affiliations:** a Institute of Digital Healthcare, WMG, University of Warwick Coventry CV4 7AL UK jerome.charmet@he-arc.ch; b 5D Health Protection Group Ltd Accelerator Building 1 Daulby Street Liverpool L7 8XZ UK; c Middle East Technical University, Mechanical Engineering Department 06800 Ankara Turkey; d Department of Analytical Chemistry, Faculty of Pharmacy, Gazi University Ankara 06330 Turkey; e School of Engineering, University of Birmingham Edgbaston Birmingham B15 2TT UK; f Computer Science Research Centre, Royal College of Art London SW7 2EU UK ali.asadipour@rca.ac.uk; g Warwick Medical School, University of Warwick Coventry CV4 7AL UK; h HE-Arc Ingénierie, HES-SO University of Applied Sciences and Art of Western Switzerland 2000 Neuchâtel Switzerland

## Abstract

Electrochemical sensing is ubiquitous in a number of fields ranging from biosensing, to environmental monitoring through to food safety and battery or corrosion characterisation. Whereas conventional potentiostats are ideal to develop assays in laboratory settings, they are in general, not well-suited for field work due to their size and power requirements. To address this need, a number of portable battery-operated potentiostats have been proposed over the years. However, most open source solutions do not take full advantage of integrated circuit (IC) potentiostats, a rapidly evolving field. This is partly due to the constraining requirements inherent to the development of dedicated interfaces, such as apps, to address and control a set of common electrochemical sensing parameters. Here we propose the PocketEC, a universal app that has all the functionalities to interface with potentiostat ICs through a user defined property file. The versatility of PocketEC, developed with an assay developer mindset, was demonstrated by interfacing it, *via* Bluetooth, to the ADuCM355 evaluation board, the open-source DStat potentiostat and the Voyager board, a custom-built, small footprint potentiostat based around the LMP91000 chip. The Voyager board is presented here for the first time. Data obtained using a standard redox probe, Ferrocene Carboxylic Acid (FCA) and a silver ion assay using anodic stripping multi-step amperometry were in good agreement with analogous measurements using a bench top potentiostat. Combined with its Voyager board companion, the PocketEC app can be used directly for a number of wearable or portable electrochemical sensing applications. Importantly, the versatility of the app makes it a candidate of choice for the development of future portable potentiostats. Finally, the app is available to download on the Google Play store and the source codes and design files for the PocketEC app and the Voyager board are shared *via* Creative Commons license (CC BY-NC 3.0) to promote the development of novel portable or wearable applications based on electrochemical sensing.

## Abstract 2

The platform-agnostic PocketEC app, optimised for electrochemical sensing assay development, can communicate seamlessly with several IC based potentiostat platforms.

## Graphical abstract

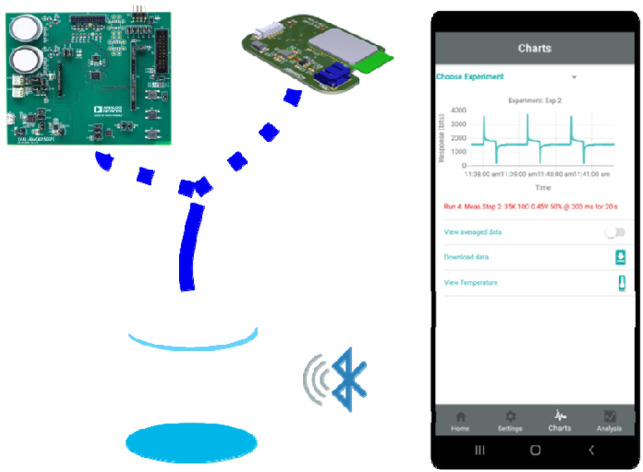

## Introduction

Electrochemical sensing represents a growing field of research that has expanded through the development of novel materials and sensing modalities. With such developments, new applications have started to emerge including for remote or on-site sensing. Applications include biosensing,^1–4^ environmental monitoring,^5–8^ or battery characterisation^9–13^ to cite a few. Such applications are now constrained by the use of expensive and bulky desktop potentiostats, usually developed and tailored for laboratory settings and that are not well suited for wearable or portable applications.

To overcome these limitations, a range of small potentiostats were reported in the literature in recent years, alongside a few commercial options. Several custom-built platforms using discrete analogue components interfaced with dedicated microcontroller units (MCU) have been reported^14–17^ and, more recently, others took advantage of commercial analogue front end (AFE) or systems on chip (SoC) potentiostat integrated circuits (ICs). These can provide advanced functionality in a much more compact format, and greatly simplify development of new solutions. Projects based around the LMP91000 AFE (Texas Instruments), an IC used in a range of commercial portable potentiostats,^16^ were reported in the literature.^18–21^ The Analog Devices ADuCM355 SoC with integrated microcontroller, which equips commercial product (see *e.g.* ref. 22), has also been exploited recently to detect aptamers/ligand binding^23^ or sweat rate and salt concentration therein.^24^ One issue with potentiostat IC is their specific communication protocols that put constraints on the development of dedicated interfaces.

Even though open source potentiostats, with wired connection, have been reported previously (*e.g.* to computers *via* serial communication^15,18,25–28^ or smartphone *via* the audio jack^29^) most applications are based on wireless communication protocols, usually with smartphones or tablets *via* Bluetooth, BLE,^14,30,31^ WiFi^16,32,33^ or NFC.^34^ Wireless communication, for control and/or data acquisition, enables small footprint, battery-operated, potentiostats to be used in enclosed spaces as well as for wearable applications.^14^ Smartphones, whose use is now widespread including in resource limited settings,^35^ are a candidate of choice due to their ever increasing computing power, the possibility to share the results quickly, and back-end processing possibilities (*e.g.*, in the cloud). Dedicated apps or web interfaces are typically used to communicate with the potentiostats. Apps provide fully integrated solutions, however their development requires a specific set of skills, and their use is typically limited to one dedicated potentiostat. Bearing in mind that most developments rely on established electrochemical sensing assays, each app proposes similar interfaces and parameters to set-up and control the assays. Indeed, the main difference between most apps lies in the communication protocol with their dedicated potentiostats. Consequently, significant development effort is lost on the (re)programming of a set of common, cross-platform settings each time a new potentiostat is proposed. This process could be greatly simplified through the development of a versatile app comprising a set of pre-programmed electrochemical settings and programmable communication protocols.

Finally, most solutions proposed to date were developed from an electrochemical research point of view and whilst providing the fundamental measurement data, stop short of the conversion to the desired result required to make the measurement accessible to a wide audience. For example, most platforms report current or voltage depending on the assay, whereas a concentration would be most appropriate for biosensing assay development.

With the above considerations in mind, we propose an integrated solution comprising of (1) the PocketEC, a universal app, that can be made compatible with a range of potentiostat chips, (2) the Voyager board, a versatile miniature potentiostat, and (3) an intermediary data communication Arduino-based platform for third-party potentiostats with buffer-stored data. The user-friendly PocketEC app, the main focus of the paper, has the following attributes: (i) it is based on a novel concept that makes it easier to use with a range of potentiostats; it uses a JSON property file to address firmware and hardware specific commands, making it usable with a range of potentiostats as demonstrated here using the custom-made Voyager board, the commercially available ADuCM355 evaluation board, and the open-source DStat potentiostat. Each board harbours unique AFEs and MCUs; Voyager board (PIC24F MCU and LMP91000 AFE) ADuCM355Eval board (ARM Cortex M3 MCU and ADuCM355 AFE), and DStat board (ATxmega256A3U MCU and discrete elements AFE) chosen to highlight the versatility of the PocketEC app. (ii) It contains a calibration procedure that can be used pre- or post-measurement to convert the results into context-relevant units such as concentration and (iii) it proposes a set of flexible parameters that allow the user to create and recall a wide range of user defined electrochemical techniques to condition electrochemical sensors and measure their responses. Finally, (iv) the app is available on the Google Play Store and its source code is available *via* Creative Common License (CC BY-NC 3.0). The small footprint (<14 cm^2^) Voyager board, based around the LMP9100 AFE (i) is battery operated, (ii) communicates with the app *via* Bluetooth and (iii) its source code and design files are made available *via* Creative Common License (CC BY-NC 3.0). The Voyager board, presented here for the first time, provides a simple interface that will work with the PocketEC app without any reprogramming.

**Fig. 1:**
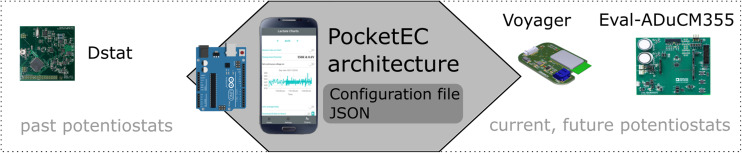
Schematic of the PocketEC app communicating, *via* Bluetooth, with MCU based potentiostats. We demonstrate communication with established platforms such as the DStat as well as a custom-made Voyager board and the commercially available EvalADuCM355 board. An Arduino board was used to act as buffer between the DStat and the PocketEC app as the latter was designed for real-time communication and the former requires a buffer.

We demonstrate the versatility of the app, by measuring an amperometric Ferrocene Carboxylic Acid (FCA) assay on the DStat potentiostat, the ADuCM355 evaluation board, and on the custom-made battery-powered Voyager board (Fig. 1). We also compare the results from the Voyager board with a commercial benchtop laboratory potentiostat (Digi-IVY 2322, US) for the FCA assay as well as for a silver assay using anodic stripping. Our results demonstrate the versatility of the PocketEC app that can seamlessly communicate with the portable potentiostats. They also show good agreement between the tabletop potentiostats and the voyager board with calibration curves exhibiting very good linearity, and <1% difference in slope.

## Methods

### App architecture and programming

The hybrid mobile app was developed using Ionic Framework 3, so that the same code for the mobile app can be used in multiple mobile platforms. The source code is available at https://github.com/Pocket-EC. The app, available on Google Play Store, was tested on several Android based smartphones and tablets from Samsung Galaxy S7 (Android 7) to Google Pixel4 (Android 12). It may also be compiled for iOS and Windows. In the case of iOS, the potentiostat must use Apple's MFi technology to connect to the phone.

### Voyager board

**Fig. 2:**
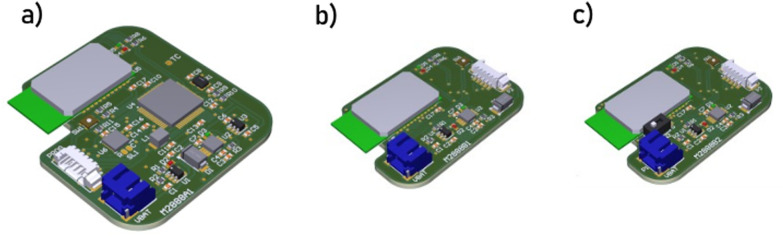
The Voyager board design variant; (a) single-sided board, (b) double-sided board, and (c) double-sided board with mode sliding switch to select between two and three electrode configuration.

The Voyager board is based around the Integrated Analog Front End (AFE) potentiostat – LMP91000 by Texas Instruments.^36^ It has an internal programmable control logic module with three control registers (CNs); TIA control register (TIACN), reference control register (REFCN), and mode control register (MODECN). An integrated temperature sensor is available to reduce noise caused by ambient temperature variations. A PIC24F microcontroller with 12-bit ADC modules was chosen to drive the LMP91000 *via* an I2C connection. This MCU is used across a broad range of industrial applications which enables enhanced accuracy in sampling, and reliable wireless communication with the app. The code was programmed in C using the MikroElektronika's integrated development environment (IDE).^37^ Three prototype boards were designed and fabricated in-house (Altium Designer) as shown in Fig. 2. The final design, reported herein is a double-sided board with mode sliding switch to select between two and three electrode configuration. The PCB layout design and firmware are available at https://github.com/Pocket-EC.

### Evaluation board

We used the evaluation board EVAL-ADUCM355^38^ by National Instruments to demonstrate the universality of the PocketEC app. It is based on the ADuCM355, an on-chip system that controls and measures electrochemical sensors and biosensors. It integrates an ARM Cortex M3 microcontroller, 16-bit 400kSPS ADC, programmable gain amplifier, antialiasing filter and AFE. The Digital-to-Analog Converters (DACs) which control the noninverting inputs of the potentiostat amplifier and the transimpedance amplifier (TIA) output voltage from 0.2 V to 2.4 V with at least 0.54 mV resolution. The TIA is suitable for measuring current output from ±0.00005 μA to ±3000 μA and the load and gain resistors of this TIA are programmable. The existence of high-speed circuitry also makes impedance measurement possible in ADuCM355 based solutions. The large output voltage range, ability to implement most electrochemical sensing modalities, highly integrated design and highly controllable circuitry makes this platform a candidate for wide implementation in research settings and in platforms requiring multi-sensing capabilities.

Functions required for control of the potentiostat and communication with the PocketEC app were implemented using the M355_ECSns_SingleWE example provided by AD^39^ as reference. This implementation allows for the setting of TIA gain resistor, load resistor, voltage bias and voltage zero values relevant for channel 0. The code is available at https://github.com/Pocket-EC.

### DStat board

The DStat board is built around the ATxmega256A3U microcontroller (Microchip Technology, San Jose CA, USA).^40^ It has a potentiostat circuit that uses a transimpedance amplifier (TIA) with programmable gain ranging from 100 Ω to 500 MΩ for current measurements. The circuit is designed with an additional unity gain amplifier, to limit the current flow through the reference electrode (RE). An external 16-bit DAC unit is integrated with a step size of 46 μV for the 0–3 V range. DStat board utilises a 24-bit ADC, which is integrated with a digital antialiasing filter for reduced noise measurements. Due to the high resolution of the ADC, the instrumental limit of detection for current measurements is approximately 600 fA. The maximum current output with the lowest gain resistor (100 Ω) is 15 mA, offering a large output range.

The potentiostat circuit can perform various types of electrochemical measurements, including chronoamperometry, cyclic voltammetry, differential pulse voltammetry, square wave voltammetry, and potentiometry. The board, initially developed for wired communication, was reprogrammed following the open access instructions,^41^ and by using the Olimex AVR ISP Mk2 external programmer to enable wireless communication. The new firmware code is available at https://github.com/Pocket-EC.

### Arduino board

We used an Arduino Mega 2560 microcontroller board as a buffer platform for the non-real time measurements of DStat potentiostat. Indeed, our app was designed to account for more demanding real-time measurements, however, consequently it cannot handle buffered data. The choice of well-established and widely available Arduino boards is to ensure an easy integration with other platforms. The Arduino integrated development environment (IDE) 1.8 was used to program the Arduino board. The Arduino sketch is available at https://github.com/Pocket-EC.

### Electrochemical sensing

#### Sensor electrodes

Electrochemical assays used commercially available gold sensor electrodes with an integral silver reference electrode and gold counter electrode (Dropsens, Metrohm, UK). Individual sensors were first prepared to ensure reproducible behaviour for experiments across different potentiostats. They were prepared by first removing traces of silver from the working electrode which was occasionally observed, presumably from the fabrication process. This was done by cycling the working electrode between −0.1 and +0.5 V at 50 mV s^−1^, five times, in 50 mM NaNO_3_ in high purity (>18.2 MΩ) water. The potential of the silver reference electrode was also observed to vary between different sensors when compared to a standard Ag/AgCl reference electrode (BAS, Metrohm, UK) or the measured FCA redox potential. This was minimised after chloridisation, which was achieved by holding the reference electrode at 0.2 V *vs.* an external Ag/AgCl reference electrode (BAS, Metrohm, UK) for 30 s in 0.1 M NaCl(aq) solution.

#### Amperometry

Ferrocene Carboxylic Acid (FCA). A stock solution of 1 mM FCA (Sigma-Aldrich, UK) in Phosphate Buffered Saline (PBS, pH 7.4) (Sigma-Aldrich, UK) was made up using deionised water. A prepared gold electrode (Dropsens, Metrohm, UK) was immersed in PBS solution, pH 7.4, and connected to a laboratory potentiostat (Digi-Ivy). The sensor performance was first checked by cyclic voltammetry in PBS and 1 mM FCA solution. Following this the electrode was transferred back to PBS and connected to the test potentiostat. The working electrode was poised at 50 mV for 20 seconds, 450 mV for 20 seconds and 50 mV for 20 seconds with respect to the reference electrode. The electrode was then held at 50 mV for 180 seconds. The solution was stirred for a minimum of 60 seconds at the beginning of the final 50 mV step. The process was then repeated ensuring that measurements were only taken after the solution had been allowed to stand for more than 60 seconds. The measurement in PBS was repeated five times to allow the baseline value to stabilise and then FCA stock solution was added to produce a 0.001 mM solution and the procedure repeated. Duplicate measurements were then taken at 0.003, 0.01, 0.03, 0.1 and 0.3 mM respectively to produce a calibration curve. The procedure was repeated with a commercial laboratory potentiostat for comparison.

#### Anodic stripping multi-step amperometry

A stock solution of silver nitrate (10 mM) in sodium nitrate (50 mM) and nitric acid (10 mM) was made up in deionised water. The solution was made up fresh and stored in the dark prior to use. A prepared gold electrode (Dropsens, Metrohm, UK) was connected to the potentiostat (Digi-Ivy or Voyager board respectively) and the electrode immersed in a solution of 50 mM sodium nitrate and nitric acid (10 mM). The following working electrode potential sequence was used: 0.5 seconds at 0 V, 50 seconds at −200 mV, 100 seconds at 0.200 mV. When integrating the charge, the 100 seconds (stripping) step at 0.200 mV was split into 5 seconds and 95 seconds, the 5 seconds measurement being used for the analysis. After repeating the blank measurement, additions of stock silver solution were added to produce silver solutions of 0.003, 0.01, 0.03, 0.1, 0.3, 1, and 3 mM respectively with duplicate measurements for each concentration. The solutions were stirred between each measurement and after each addition and allowed to settle for at least a minute between measurements. The data were used to construct a calibration curve for silver.

## Results and discussion

### PocketEC app and communication protocol

#### PocketEC app

**Fig. 3:**
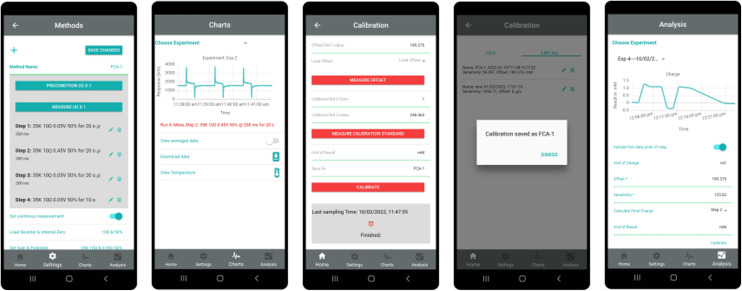
The PocketEC app proposes a flexible interface to communicate with a range of potentiostats and enables the creation and recall of multi-techniques conditioning and measurement steps. Screenshots of the main features with the Methods, Charts, Calibration and Analysis sections.

The PocketEC app was designed to provide a simple user experience. It comprises three sections for (1) controlling the device (settings), (2) real-time visualisation of measurements (charts) and (3) processing of stored data (analysis). Fig. 3 shows selected screenshot of each module. In the settings module, the user (1) configures the app to communicate with the potentiostat by setting firmware specific communication parameters and (2) defines commands to be sent *via* Bluetooth Serial communication to preconfigure the device with the experimental parameters. Upon reception, the device starts the measurements and sends the data points back to the app, which stores them with a timestamp. The frequency at which the data is sent back is defined by the user and can therefore allow for near real-time visualisation. This feature facilitates new experiment designs and troubleshooting. Temperature is recorded at the end of each measurement cycle, allowing for temperature-dependent drifts to be accounted for.

**Fig. 4:**
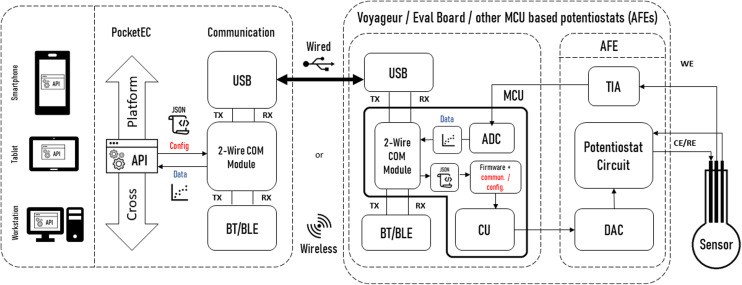
Typical architecture of a complete system for wired and/or wireless solutions. The parts in red indicate the platform dependent configuration and communication classes that need to be implemented.

Importantly, the app provides all the functionality required to communicate with a range of MCU-based potentiostats. This unique feature is enabled by the provision of a universal editable property file that defines the communication rules between the app and potentiostat. This user-editable file is all that is needed on the app side to enable communication with the potentiostats. A sample property file, specified in JavaScript Object Notation (JSON) – a standard text-based format for representing structured data, is available at https://github.com/Pocket-EC. On the MCU code, it suffices to add a configuration and communication class. Fig. 4 shows the typical architecture of a complete system for wired and/or wireless solutions. The communication is independent from the AFE or MCUs used since all wired or wireless solutions use the 2-wire serial communication module (TX/RX). The only parts that need to be edited to allow full communication are shown in red.

#### Communication protocol

The app communicates with the potentiostat *via* Bluetooth. To start using a new potentiostat with the app, the user needs to perform a one-time process of linking the potentiostat to the app. This is done in the app's SETTINGS (Fig. 3a) by scanning for devices. Once a link is established the app is configured to communicate with the potentiostat in two stages. The first stage sets up fundamental rules of two-way communication between the app and the potentiostat. This is as specified by the properties in the JSON file described earlier. The second stage configures the measurement cycle required for running an electrochemical assay, which can be made up of one or multiple electrochemical techniques. Each technique specifies the voltage to be applied to the potentiostat, duration of applied voltage and the frequency at which the generated output voltage is sampled. All experimental parameters required for an assay can be saved as a Method and can be referenced again later. Multiple methods can be created and stored in the app and old methods can be edited and reused (see Fig. 3).

Once a method is created, an experiment can be started from the app. The app communicates with the potentiostat and sends commands as set in the Method by the user. At user specified intervals, the app requests data from the potentiostat which are stored on the phone with a timestamp. The collected data are processed and are available to view in near real-time. Compared with other approaches that rely on sequential communication, where the full protocol is typically sent to the potentiostat followed by the resulting timeseries being returned to the app at the end of the experimental run, we have opted for an approach allowing for near real-time monitoring. While this approach is particularly well suited for assay developments as it enables seamless troubleshooting and fine-tuning, it comes with its own challenges. As an example, the AduCM355 includes three sequencers that can be used to program instruction sets. Although this strategy provides good control over the experimental timing it also results in an overhead during development. Indeed, an intermediate representation of the protocols must be created, sent to the potentiostat device, and interpreted before its execution.

To avoid this, the PocketEC app is responsible for timing experimental steps, sending the minimal viable instruction set for execution of a particular experimental step in the potentiostat. In a reasonable experimental setup where the Bluetooth module of the host device is not under significant traffic, the delays introduced between instructions produced by the app and received by the potentiostat's internal logic should be consistent and will not introduce any significant error in the data collected.

Another unique feature of the app is the *Calibration module* that enables the user to view the data in application-specific units such as concentration. This feature, also available in real-time, is achieved *via* manual entry of sensitivity and offset parameters, or *via* on-board calibration measurement. This is particularly useful for non-experts who may struggle to make the link between the data and the expected measurement otherwise. Data analysis can also be carried out externally. Options to export the saved data in CSV (comma separated values) format by email, Bluetooth and other sharing methods are available. The detailed communication protocol and measurements steps are available at https://github.com/Pocket-EC.

### Voyager board

**Fig. 5:**
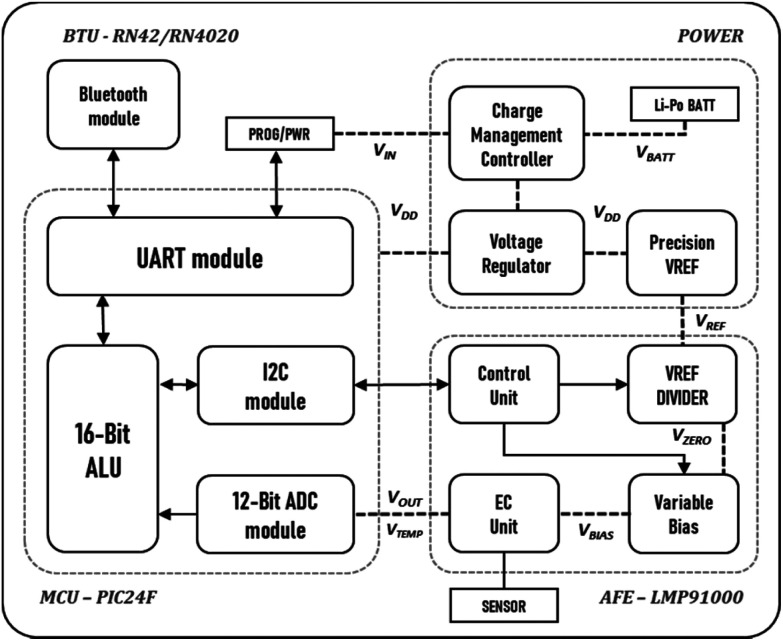
Voyager board block diagram.

Key design requirements were identified to prototype a preliminary solution (hardware and software), test its usability in a lab setting, and refine it in a circular design as detailed below. We first designed a one-sided board with a Bluetooth module (RN42APL by Microchip Technology^42^) shown in Fig. 2a as a proof-of-concept prototype. Following successful evaluation (data not shown), we designed and fabricated a smaller footprint option consisting of a double-sided board (Fig. 2b). Switches and configurable components added to the third version (Fig. 2c) enable the board to be used in different electrochemical or biomedical scenarios. The general block diagram of the Voyager boards is shown in Fig. 5. The chip was programmed in-house to provide remote configuration and various data transfer modes (*e.g.*, on-demand, burst, and pre-defined intervals) over wireless communication. The source code is available at https://github.com/Pocket-EC. Lithium Polymer batteries (Li-Po) were purchased with different capacities to test and compare them against the simulated results outside a lab setting (experimented). The Voyager v3.0 is a small footprint board suitable for wearable applications. The source code, design files and bill of materials are available at https://github.com/Pocket-EC.

### Evaluation board

Implementation of a new sensing platform is likely to make use of stable evaluation platforms provided by the IC manufacturer at early stages. However, these evaluation platforms are typically designed for wired communication with a computer-based interface, being a limited proxy for the development of solutions that require and are limited by a wireless communication protocol, and that communicate with a mobile app. To validate our approach, we have selected the ADuCM355 evaluation board.

To communicate with the PocketEC app, a HC-06 Bluetooth module was interfaced to the evaluation board by removing the jumpers (JP45 and JP46) connecting the AduCM355 and UART-to-USB ICs and connecting the Rx and Tx signals to the HC-06 board. The BT module was powered *via* the JTAG connector header. The module's baud rate was changed to match the AduCM355's using AT commands provided by the manufacturer. By using a module capable of transparent UART communication, minimal setup was necessary. The approach would be similar upon future implementation of a BLE solution. No significant delays in communication were observed when compared with the Voyager board.

### DStat potentiostat

To further validate the versatility of the PocketEC app, interface it with DStat, a well-established MCU-based open-source potentiostat.^26^ Initially designed to interface with a custom computer-based platform using wired communication (USB), DStat required modifications to enable seamless communication with a mobile app *via* Bluetooth. This was achieved by replacing USB communications device class (CDC) with USARTC0 port in XMEGA MCU to enable serial communication *via* Rx/Tx signals.

**Fig. 6:**
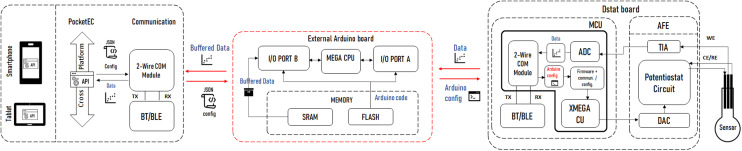
Block diagram for DStat measurements setup. The red areas indicate the additions made to the previous architecture.

The PocketEC app was designed to enable near real-time communication, which is fundamentally different from the DStat communication protocol designed to receive all the experiment specifications before execution. To address this disparity, an intermediate Arduino-based platform was integrated into the system. The platform was programmed to receive the complete experimental parameters protocol sent by the PocketEC app and subsequently relay it to the DStat. Additionally, the platform acts as a buffer to store the measurements acquired by DStat and transmitting them individually to the PocketEC app upon request. Arduino Mega board^43^ with 4 UART hardware ports was utilised for the two serial communications. Specifically, Tx1/Rx1 pins were connected to UART port C on DStat board, and Tx2/Rx2 were dedicated to the HC-06 Bluetooth module. Like with the ADuCM355 evaluation board and the Voyager board, no significant delay was observed upon connection. Fig. 6 shows the established architecture created for DStat measurements.

The Arduino board was also programmed for data processing including the conversion of ADC and DAC units to desired amperes and volts units. These fundamental conversions were originally applied on the computer-based interface developed for DStat. However, to minimise the changes on the app settings, they were applied on the Arduino platform. The Arduino board was further programmed to address the difference in time intervals for measurements. While this parameter is a user-defined value that determines the frequency at which data is sent back to the app, this value is set by the DStat ADC sampling rate. The Arduino board was programmed to send the average of several subsequent data points based on the user-defined value, thus bringing the time intervals into close agreement.

This novel integration between the PocketEC app and DStat potentiostat showcases their compatibility and establishes a seamless connection for efficient potentiometry analysis. This near real-time measurement setup, using and Arduino platform, was created to overcome the substantial disparities in communication protocols. By implementing this approach, similar adaptations can be effortlessly applied to other potentiostats that rely on receiving complete experiment parameters prior to execution. This implementation ensures compatibility without necessitating reprogramming on either the app or a potentiostat, making it a flexible and scalable solution for diverse potentiometry applications.

### Overview of the communication with different platforms

Above, we have seen how the PocketEC app can be interfaced with different potentiostats. This was done without any reprogramming of the app, thanks among others to its JSON configuration file. This file is used to set up fundamental rules of two-way communication between the app and the potentiostat; format of transmission of commands from app to the potentiostat, expected format and length of data received from potentiostat and listing of all possible values that can be used to program the control registers of the potentiostat. The properties in the JSON file are described in Table 1. The app contains a list of JSON properties with default values. These properties can be edited by the user *via* SETTINGS and set with potentiostat dependent values. These properties need to be set just once for every potentiostat or whenever there are firmware updates in the potentiostat. Multiple JSON configurations can be created and stored in the app. Sample JSON files are provided for each of the 3 potentiostats used here. After this initial communication configuration step, the configuration of the potentiostats, as described above, can take place.

**Table 1:** JSON configuration parameters

Electrochemical relevance	Variable/property name	Comments	Example values
Gain (used to define the current range)	TIACN control register	Transimpedance amplifier resistor based on the values dependant on the AFE circuit	• Voyager board, 2.75 KΩ to 350 KΩ, 35 KΩ used for CA measurements
			• EvalADuCM355, 0 to 512 KΩ range, 32 KΩ used for CA measurements
			• DStat, 100 to 100 M range, 30 KΩ used for CA measurements
Load resistance	TIACN control register	Programmable resistive load that also affects the gain	• Voyager board, 10 Ω to 100 Ω range, 10 Ω used for CA measurements
			• EvalADuCM355, 0 to 3.6 KΩ range, 10 Ω used for CA measurements
			• DStat, not defined
Applied voltage	REFCN control register	Experiment-dependant bias voltage applied to the electrochemical cell at each step	• Voyager board, ±0.6 V at 0.05 V intervals with programmable percentage of the source reference (20%, 50%, 67%)
			• EvalADuCM355, ±0.6 V at 0.05 V intervals
			• DStat, ±1.5 V range
Voltage duration	Duration (s)	Duration of the applied voltage at each step	• Experiment-dependant. Default value 3 s in the Methods section
Operational mode	MODECN control register	Defined to set the board in a potentiostat 3-lead mode	• Voyager board, this value is set to 3 by default
			• EvalADuCM355, 3
			• DStat, not defined
Sampling Rate	Data frequency (ms)	Defines how fast the potentiostat collects measurement values	• Voyager board, this value is set to 200 ms by default
			• EvalADuCM355, 200ms
			• DStat, ∼200 ms adjusted by averaging
Temperature sensor	readTemperature property	Integrated temperature sensor measurements	• Voyager board, temp value sent in bytes at the end of each step
			• EvalADuCM355, temp values sent in string
			• DStat, not integrated

As mentioned above, although the PocketEC app does not require additional changes, some programming adjustments are necessary on the potentiostats’ side to accommodate for microcontroller-specific settings. However, using manufacturers recommended codes (see *e.g.*, ref. 39 for the AduCM355), it becomes feasible to address the required registers for real-time measurements. To facilitate buffered communication, we have proposed to use an Arduino board to handle the register addressing buffering. Table 2 shows the main parameters for managing these settings with corresponding examples for three potentiostats that were connected to the PocketEC app in this study.

**Table 2:** . Communication protocols

Property name	Description	Example values
responseDelimiter	Expected string at the end of all communications from Potentiostat to app when format of communication is String	"\r\n”
Test	The property value pairs included in this property specify communication rules between app and Potentiostat in Test mode when the Potentiostat is linked to the app the first time.	"Tx”: ““
		“TxAsString”: true
		“RxAsString”: true
		“RxLength”: “3”
readData	The property value pairs included in this property specify communication rules between app and Potentiostat for reading data.	"Tx”: “3”
		TxAsString”: true“
		“RxAsString”: false
		“RxLength”: “3”
readTemperature	The property value pairs included in this property specify commands and communication rules and between app and Potentiostat for reading temperature.	"Tx”: “2”
		“TxAsString”: true
		“RxAsString”: true
		“RxLength”: null
setConfigMode	Specifies the command required for setting the Potentiostat into configuration mode where it expects the next command to contain commands for setting its registers. In this example ‘0’ sets the potentiostat in config mode. TxAsString should be set to true if app sends the command ‘0’ as a string to the potentiostat. RxAsString should be set to false if expected reply should be read as bytes. Rx specifies expected reply from the Potentiostat and RxLength the length of the expected bytes in the reply	"Tx”: “0”,
		“TxAsString”: true,
		“RxAsString”: false,
		“Rx”: “Send Three Bytes”
		“RxLength”: “18”
setRegisters	This property defines the communication format and expected reply when the app sets the registers of the potentiostat	"Tx”: null
		“TxAsString”: false, “RxAsString”: false
		“Rx”: “TIACN OK\r\nREFCN OK\r\nMODECN OK”
		“RxLength”: “31”
REFCN	Lists all values that can be used to set the Reference control register	{"description”:"20%”, “SETTING”: [{"description”:"0.6 V 20%”,"command”:"157”},]}

### Electrochemical measurements

For the app and board development, the decision was taken to read the raw data from the board and process it in the app. This is to minimise the level of computation and data analysis required on the board, which will help minimise the complexity and cost of development. One consequence of this however is that where the data acquired is time dependent, this needs to be accounted for. For the Voyager board once a potential is applied, the ADC is read continuously, dictated by the microcontroller duty cycle, and the result added to a running tally. When the value is read by the app, it transfers the average value and the number of times the ADC has been read. So, the value read represents an average current (bits) value over the whole-time interval since the last reading, rather than an instantaneous value which could vary according to differences in the exact timing. This is especially important where, for example, the current passed was decaying significantly in the time-period following a potential step, as is often the case.

During the analysis, where the charge passed is calculated from the data acquired during a defined amperometric measurement step, the actual accumulated time for the step can vary by up to the length of the repeat time. This is accounted for by scaling the time interval and charge of the last point to ensure that the calculated charge is measured over the same total time period.

The applicability of the app across multiple development boards was demonstrated using an amperometric measurement of the redox mediator FCA in PBS. Data from the Voyager, ADu, and DStat boards were compared with data from a commercial laboratory potentiostat, shown below. The current sensitivities for the Voyager board were calibrated using an external dummy cell with selectable resistors.

#### Amperometry

**Fig. 7:**
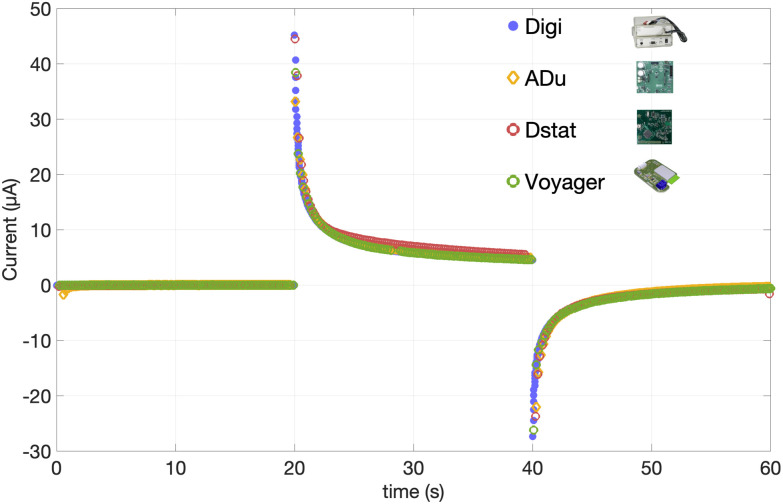
Amperometric measurement for 1mM FCA in PBS. Voltage applied 0.05 V 0–20 s, 0.45 V 20–40 s and 0.05 V 40–60 s. Measurements with commercial laboratory potentiostat (Digi), Voyager board, DStat board, and ADuCM355 evaluation boards (ADu). Because the acquisition rate is lower for the development boards, the initial datum at each transition is offset to the mid time-point of the acquisition.

Fig. 7 shows the oxidation current passed during an amperometric *I*–*t* measurement for the Au electrode in a 1 mM FCA stock solution, at 0.45 V for 20 s, followed by a step to 0.05 V for 20 s. The data from the Voyager, ADU, and DStat boards (200 ms interval) are superimposed on the data from the commercial potentiostat (interval 20 ms) and show very good correlation.

**Fig. 8:**
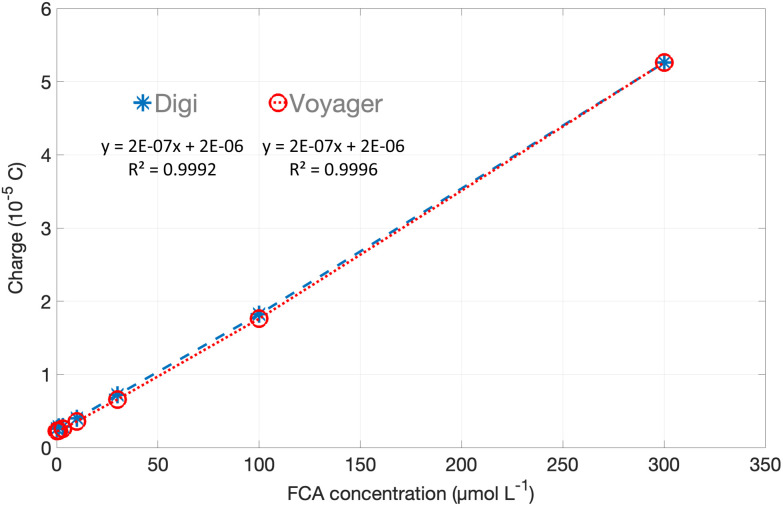
Calibration assay for FCA. Charge *vs*. concentration, 0 to 300 μM FCA in PBS using amperometric measurement shown in Fig. 6. The error bars show the range of the duplicate measurements. They are within the size of the markers, so are plotted but not visible.

To compare differences between the measurements obtained with the app/development boards and a laboratory potentiostat, more detailed comparisons were made using the Voyager board. The FCA stock solution and the same measurement procedure was used to create a calibration curve for FCA in PBS, shown in Fig. 8. Here the charge passed during the 20 s oxidative step was calculated for each measurement. The two data sets are barely distinguishable, with <1% difference in the slope, demonstrating the performance of the Voyager board.

#### Anodic stripping

The Voyager and Digi-Ivy potentiostats were also compared for an Ag anodic stripping multi-step amperometry measurement. This is a more challenging situation, as Ag is collected on the working electrode during an extended reduction phase, traditionally accompanied by rapid stirring, and is measured by reoxidising the deposited Ag, which occurs as a high current pulse over a short period of time. Using the relatively large DropSens Au sensor electrodes, this current saturated the range of the Voyager board.

**Fig. 9:**
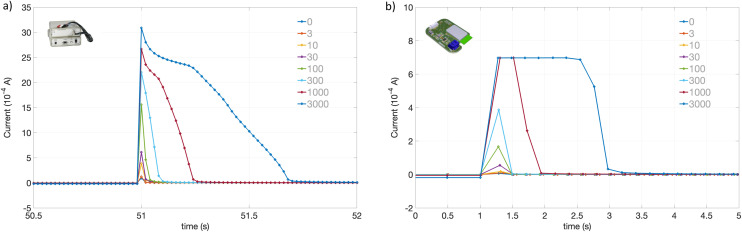
Silver anodic stripping. (a) Silver anodic stripping step *vs.* time for 0 to 3000 μM AgNO_3_ using commercial laboratory potentiostat, acquisition time 20 ms. (b) Silver anodic stripping step *vs.* time for 0 to 3000 μM AgNO_3_ using Voyager board. Acquisition time 200 ms, 2.7 kOhm range, 100 Ohm load resistor.

The overlaid anodic stripping currents are shown in Fig. 9. In Fig. 9a, using the laboratory potentiostat we can see that up to 300 μM Ag^+^, the oxidative charge is passed within 100 ms. After this the time taken extends as more than a monolayer of Ag^0^ is deposited and this slows the oxidation process down.

Fig. 9b shows the equivalent data obtained with the Voyager board. As the acquisition interval is 200 ms, this is slower than the oxidation current spike, however the average value is captured by the faster on-board measurement and integration. As the current has saturated, the current is passed over a longer period of time for the higher Ag^+^ concentration values. This takes 2 s at 3 mM Ag^+^, compared to ∼0.7 s with the laboratory potentiostat.

**Fig. 10:**
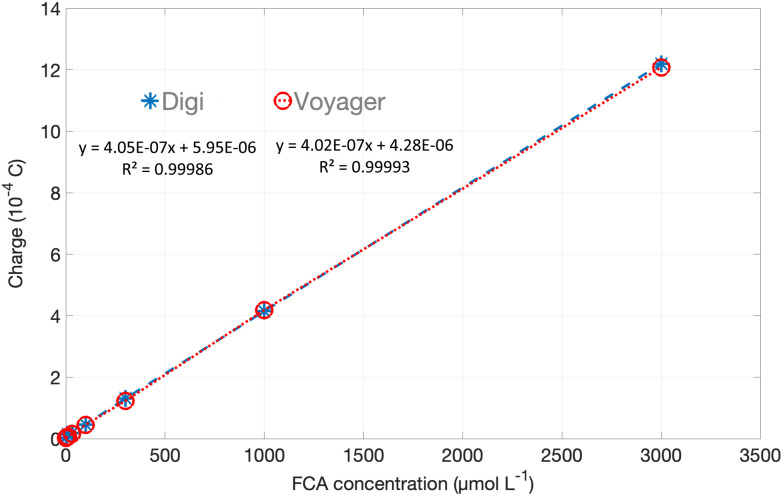
Silver anodic stripping calibration plots for commercial tabletop potentiostat (Digi) and Voyager board. Integrated charge *vs.* Ag^+^ concentration. The error bars show the range of the duplicate measurements. They are within the size of the markers, so are plotted but not visible.

The calibration curves for the two potentiostats are overlaid in Fig. 10, and both show very good linearity with <1% difference in slope between the two devices. Overall, the comparison between measurements using the Digi-Ivy potentiostat and Voyager boards show very good agreement even where the timescale of the app acquisition or the current range of the Voyager board are limited. It should be noted that this has relied on pretreatment of the Au sensors to make their performance sufficiently reproducible.

## Conclusions

In this manuscript, we have presented the PocketEC, a platform-agnostic app tailored for wearable or portable electrochemical sensing applications. The app, optimised for assay development, can communicate seamlessly with several IC based potentiostat platforms as facilitated by a user-defined property file to address firmware and hardware specific commands. The advantage of such an approach is that the app can be used with several potentiostat ICs, including future generation of ICs with minimum programming investment.

The versatility of the app was demonstrated by interfacing it with three significantly different MCU based potentiostats, namely the commercially available ADuCM355 evaluation board, the open-source platform DStat, and the Voyager board, a custom-built companion board presented here for the first time. The boards were evaluated against a tabletop potentiostat by measuring an amperometric Ferrocene Carboxylic Acid (FCA) assay and a silver assay using anodic stripping. The results have shown excellent agreement, with calibration curves exhibiting good linearity and less than 1% difference in slope.

The PocketEC is available to download on the Google Play Store and the source codes for the app and the boards (Voyager, ADuCM355EVAL, and Arduino) are provided alongside the design files for the Voyager board. It is also noted that the PocketEC app can be compiled for iOS devices using Apples’ MFi technology or for Windows Mobile. We anticipate that the results presented herein will promote the development of portable and wearable electrochemical sensing applications in fields including *in situ* battery monitoring, wearable health monitoring devices or remote sensing applications to name a few.

## Author contributions

Conceptualisation: NF, MS, JC. Investigation, methodology: VM, RR, SS, AA, BD, UT. Validation: MS, NF, EY. Software: VM, RR, AA. Supervision, TA, JC. Funding acquisition TA, AA, UT, EY, NF, DI, JC. Writing – original draft: RR, VM, SS, AA, MS, JC. Writing – review and editing all.

## Conflicts of interest

There are no conflicts to declare.

## Data availability statement

Source code for the PocketEC app, source code for the Voyager board, source code for the ADuCM355 board, design files and bill of materials for the Voyager board, a sample priority file and app screenshots are available at https://github.com/Pocket-EC

## Supplementary Material
